# Hippocampal Avoidance in Multitarget Radiosurgery

**DOI:** 10.7759/cureus.15399

**Published:** 2021-06-02

**Authors:** Zachary Gude, Justus Adamson, John P Kirkpatrick, William Giles

**Affiliations:** 1 Radiation Therapy, Duke University, Durham, USA; 2 Radiation Oncology, Duke University Medical Center, Durham, USA; 3 Radiation Oncology, Duke University Health System, Durham, USA

**Keywords:** radiosurgery, hippocampus, hippocampal avoidance, hd mlc leaves, simt, vmat, brain metastases

## Abstract

Brain metastases are a common complication for patients diagnosed with cancer. As stereotactic radiosurgery (SRS) becomes a more prevalent treatment option for patients with many brain metastases, further research is required to better characterize the ability of SRS to treat large numbers of metastases (≥4) and the impact on normal brain tissue and, ultimately, neurocognition and quality of life (QOL). This study serves first as an evaluation of the feasibility of hippocampal avoidance for SRS patients, specifically receiving single-isocenter multitarget treatments (SIMT) planned with volumetric modulated arc therapy (VMAT). Second, this study analyzes the effects of standard-definition (SD) multileaf collimators (MLCs) (5 mm width) on plan quality and hippocampal avoidance.

The 40 patients enrolled in this Institutional Review Board (IRB)-approved study had between four and 10 brain metastases and were treated with SIMT using VMAT. From the initial 40 patients, eight hippocampi across seven patients had hippocampal doses exceeding the maximum biologically effective dose (BED) constraint given by RTOG 0933. With the addition of upper constraints in the optimization objectives and one arc angle adjustment in one patient plan, four out of seven patient plans were able to meet the maximum hippocampal BED constraint, avoiding five out of eight total hippocampi at risk. High-definition (HD) MLCs allowed for an average decrease of 29% ± 23% (p* *= 0.007) in the maximum BED delivered to all eight hippocampi at risk.

The ability to meet dose constraints depended on the distance between the hippocampus and the nearest planning target volume (PTV). Meeting the maximum hippocampal BED constraint in re-optimized plans was equally likely with the use of SD-MLCs (five out of eight hippocampi at risk were avoided) but resulted in increased dose to normal tissue volumes (23.67% ± 16.3% increase in V50%[cc] of normal brain tissue, i.e., brain volume subtracted by the total PTV) when compared to the HD-MLC re-optimized plans. Comparing the effects of SD-MLCs on plans not optimized for hippocampal avoidance resulted in increases of 48.2% ± 32.2% (p* *=* *0.0056), 31.5% ± 16.3% (p* *= 0.024), and 16.7% ± 8.5% (p* *= 0.022) in V20%[cc], V50%[cc], and V75%[cc], respectively, compared to the use of HD-MLCs. The conformity index changed significantly neither when plans were optimized for hippocampal avoidance nor when SD-MLC leaves were used for treatment. In plans not optimized for hippocampal avoidance, mean hippocampal dose increased with the use of SD-MLCs by 38.0% ± 37.5% (p* *= 0.01). However, the use of SD-MLCs did not result in an increased number of hippocampi at risk.

## Introduction

Metastatic brain tumors are the most common intracranial neoplasm in adults [1]. These metastases are frequent complications from other primary cancer sites. Considering both primary and secondary cases, around 170,000 patients receive this diagnosis of metastatic brain tumors in the United States each year [2].

Brain metastases often result in a significant decrease in cognitive ability and/or quality of life (QOL). While treatments are rarely curative, adequate tumor control is imperative in improving and extending the life of patients [3]. Typically, treatment options consist of surgery, whole-brain radiotherapy (WBRT), stereotactic radiosurgery (SRS), or some combination. The combination of WBRT and SRS provides excellent tumor control, but the use of WBRT often results in reduced neurocognitive function and QOL [4-6]. For this reason, SRS alone is often a preferred method of care for patients with limited brain metastases (typically between one and four metastases) [4].

One challenge of SRS is the extensive treatment time required for patients with more than four metastases [7]. A current solution to this problem is single-isocenter multitarget (SIMT) radiation therapy that allows for an efficient treatment of multiple metastases simultaneously [7]. Additionally, planning SIMT treatments with volumetric modulated arc therapy (VMAT) maintains similar dose conformity and critical structure avoidance seen in traditional SRS [7-9].

The high doses associated with SRS techniques make it imperative to quantify dose distributions in the brain and the associated neurocognitive effects. The hippocampus is crucial in the development of episodic memory, and memory retention has a strong correlation to QOL [10]. It has been shown that for patients receiving whole-brain therapy (WBRT), deterioration of neurocognitive function (NCF) is significantly reduced when hippocampal avoidance techniques are employed [11]. These studies suggest the hippocampus could also be a potentially important structure to avoid when planning SRS treatments if QOL is concerned. This paper explores the feasibility of meeting hippocampal dose constraints for SIMT SRS planned with VMAT.

SRS treatments using VMAT are commonly performed using specialized MLCs with smaller leaf widths. These MLCs have been shown to provide better conformity for single-target SRS [12-14]. However, not all of the benefits of smaller MLC leaves realized for single-target SRS have been recognized in SIMT [15,16]. Herein, the effect of small and large MLCs on hippocampal avoidance in SIMT is investigated.

In this study, we build upon previous work in evaluating hippocampal avoidance in SIMT SRS for multimet cases [17] as well as previous work in evaluating the effects of SD-MLCs during multimet SRS treatments [16]. This study serves to both validate the prior findings of these works using an independent population and combine these analysis techniques to explore SD-MLC leaf effects in the context of hippocampal avoidance during SIMT SRS treatments planned with VMAT. Furthermore, this work was presented at the Southeast Chapter of the American Association of Physicists in Medicine (SEAAPM) General Trainee session in January, 2021.

## Materials and methods

Patient data and constraints

The patient cohort in this study comprises 40 patients enrolled in an IRB-approved protocol (Duke Health Pro00081642). Inclusion criteria for the patients considered in this study included a contrast-enhanced MRI showing greater than or equal to four treatable brain metastases, with the largest lesion being ≤4 cm in diameter. Patients need to be at least 18 years of age, have at least three months of life expectancy, and score at least a 70 in the Karnofsky Performance Status (KPS). Postoperative patients with resected brain metastases were eligible as well as those who have received previous cranial SRS/WBRT as long as the treatment was more than three months prior to the SIMT. The exclusion criteria for this study included pregnant women, patients unable to receive an MRI, or those who have greater than 10 brain metastases (excluding previously treated stable metastases). Patients were also excluded if a primary lesion with radiosensitive histology, metastases within 5 mm of the optic apparatus, or evidence of leptomeningeal disease was present. Each patient of this study was treated with a SIMT plan generated with VMAT. According to protocol, each metastasis was treated with 20 Gy in one fraction or 25Gy in five fractions if the combined planning target volume (PTV) exceeded 20 cc. There was one exception of a single target in a patient who was prescribed 18 Gy due to proximity to the brainstem. Each patient was treated on a TrueBeamSTx (Varian Medical Systems, Palo Alto, CA) with HD-MLC (2.5 mm central leaf width) with 6XFFF beam energy.

The hippocampi for each patient were retroactively contoured by a board-certified radiation oncologist, and the dose to each hippocampus was evaluated. Hippocampal dose constraints were given by the Radiation Therapy Oncology Group (RTOG) 0933 as a maximum dose at any point (Dmax) of 16 Gy and a maximum dose to cover 100% of the hippocampus (D100) of 9 Gy in a 30 Gy total 10 fraction WBRT treatment [11]. Using an alpha/beta ratio of 2 Gy, constraints were converted to one and five fractions according to the biologically effective dose (BED). The hippocampal dose constraints for D100 and Dmax were calculated to have a BED of 13.1 Gy and 28.8 Gy, respectively. Constraints for the total dose delivered were calculated to be 4.21 Gy and 6.65 Gy, respectively, for the one-fraction cases and 7.48 Gy and 12.70 Gy, respectively, for the five-fraction cases. If a treatment exceeded either of these constraints, it was replanned with the objective of meeting hippocampal constraints without compromising the other organs at risk (OAR) and PTV constraints given in the original plans.

Hippocampal avoidance with HD-MLCs

The first objective was to describe the feasibility of meeting hippocampal dose constraints while maintaining PTV coverage. This was done by retroactively replanning the originally delivered patient plans. All replans were done in Eclipse 15.6 (Varian Medical Systems, Palo Alto, CA), using a 1 mm isotropic grid size and AAA version 13.6.23 as the dose calculation model. Most replanning consisted only of adding upper objectives to the hippocampal structures; however, in some instances, adjustments to arc orientations were made when necessary to meet constraints. When hippocampal constraints were not able to be met, the planning objective shifted to reducing the mean dose to the hippocampus.

Replans were normalized such that the minimum coverage for any target was equal to the minimum coverage for any target in the original plan. Specifically, replans were normalized such that the target for which the smallest percent volume receives the prescription dose in the original plan had the same percent volume in the new plan.

HD-MLC vs SD-MLC plans

The second objective of this study is to investigate the feasibility of using SD-MLC leaves for meeting hippocampal dose constraints and the associated dose-volume effects on SIMT treatments generated with VMAT. Using Eclipse 15.6, treatments were replanned using an accelerator model that was identical to the original treatment machine differing only in MLC leaf width. HD-MLCs are 2.5 mm thick, whereas SD-MLCs are 5 mm thick.

Both the clinically delivered plans (i.e., no hippocampal optimization) and plans optimized for hippocampal avoidance were replanned with SD-MLCs. Optimization objectives were held constant when changing MLC leaf width in the clinically delivered plans. Adjustments to the optimization objectives were permitted when changing MLC leaf widths in the hippocampal avoidance plans if BED constraints were not met. The optimization process used a multiresolution (MR) model to define MLC apertures and arc fields of a treatment [18]. It begins at MR level 1 as a coarse approximation of angular dose, which becomes a finer approximation as it progresses to MR level 4 [18]. As dose calculations become finer, the model becomes less flexible to changes in dose-volume objectives [18]. Adjustments made to optimization objectives were only defined at MR level 4 to minimize effects on the plan.

All plans were normalized such that the minimum coverage for any target was equal to the minimum coverage for any target in the original clinically delivered plan.

Treatment plan evaluation

Conformity index, V20%[cc], V50%[cc], V75%[cc], and D50%[cGy] were recorded to quantify the effects of both hippocampal avoidance and the use of SD-MLC leaves on the clinically delivered treatment plans in this data set. Conformity index (CI) is defined as:

$CI = \frac{V100\%[cc]}{V(PTV)[cc])}$

where V100%[cc] is defined as the volume that is receiving 100% of the prescribed dose, and V(PTV)[cc] is the volume of the combined planning target volume.

From this definition, a perfectly conformal treatment will have a CI of 1. V20%[cc], V50%[cc], and V75%[cc] are defined as the volume of healthy tissue receiving 20%, 50%, and 75%, respectively, of the prescribed treatment dose. D50%[cGy] is defined as the minimum dose delivered to 50% of healthy brain tissue. Healthy brain tissue volume is defined as the total brain volume subtracted by the total volume of all PTVs in a treatment.

Plan types were compared with average percent change, standard deviations of that percent change, and the associated p values. Percent change is defined as:

$\%\ Change = \frac{Final - Initial}{Initial} \ast 100\%$.

An increase in a value would result in a positive value for percent change. The average percent change was calculated by adding the percent change between two plan types for each patient and then dividing by the number of patients. The standard deviation was calculated from the percent change values between two plans for each patient. p values were calculated from a two-tailed T-test based on the difference between two plan types for each patient.

## Results

Hippocampal avoidance with HD-MLCs

All 40 clinically delivered patient plans met the RTOG 0933 hippocampal constraint on D100 (BED = 13.1 Gy). Seven of the 40 plans (17.5%) did not meet the Dmax constraint (BED = 28.8 Gy). Six plans had one hippocampus that exceeded the Dmax constraint, and one plan had both hippocampi exceeding the Dmax constraint (eight total hippocampi at risk).

Three of the seven patient plans that initially exceeded the maximum hippocampal BED limit could be replanned to meet this constraint with only the addition of upper objectives on hippocampal dose. A fourth patient plan met the constraint when replanning included both additional upper objectives on hippocampal dose and an adjustment in arc geometry for one arc. In total, four cases were able to be replanned for successful hippocampal avoidance (five out of eight hippocampi at risk met BED constraints). The remaining three patient plans were unable to meet the hippocampal max BED constraint without sacrificing PTV coverage.

**Figure 1:**
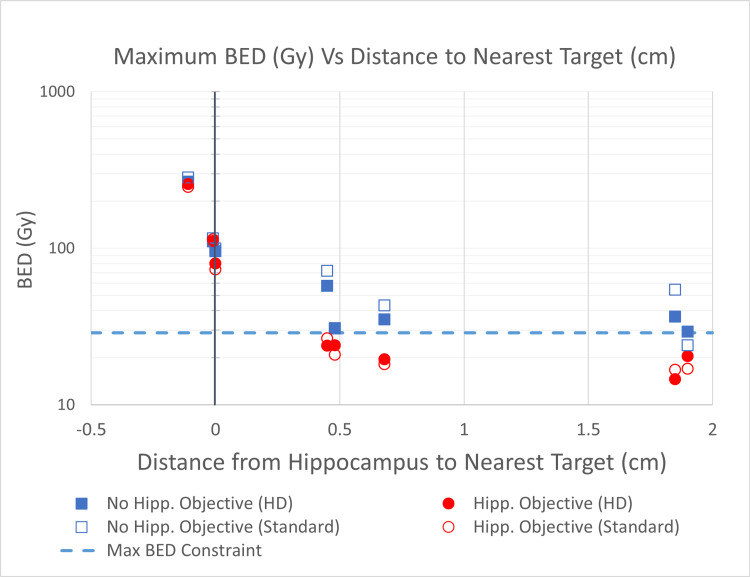
Maximum BED (Gy) vs Distance to the Nearest Target (cm) This figure shows the maximum BED delivered to each of the eight hippocampi at risk as a function of the distance between that hippocampus and the nearest PTV. Negative values of distance represent the amount of overlap between the hippocampus and PTV. All four plan types are displayed. The dotted line represents the RTOG 0933 maximum BED constraint. BED, Biologically effective dose; PTV, planning target volume; RTOG, Radiation Therapy Oncology Group.

The ability of a plan to meet the hippocampal dose constraints was directly related to the distance between the hippocampus and the nearest PTV. In the three plans that did not meet the hippocampal constraints, the nearest PTV was either touching or overlapping the hippocampus. In the four successfully optimized patient plans, there was at least 0.45 cm between the hippocampus and the nearest PTV. The minimum distance required for successful avoidance cannot be concluded from this data set; however, the trend of increasing maximum BED with decreasing distance is shown in Figure 1. This graph shows the hippocampal maximum BED of all eight hippocampi at risk as a function of distance to the nearest target. It includes the plan types of those with and those without hippocampal dose objectives (clinically delivered and replanned, respectively), each with both HD and SD-MLC leaves, making four total data points for each hippocampus at risk.

Replanning treatments for hippocampal avoidance with HD-MLC leaves resulted in an average decrease of 29% ± 23% in maximum BED delivered to the eight hippocampi at risk (p = 0.007). This data includes the three hippocampi, which were unable to meet the maximum BED constraint. If only considering the five hippocampi that were successfully replanned, then maximum BED decreased an average of 46% ± 17% (p = 0.01).

There was no statistically significant change in V20%[cc], V50%[cc], V75%[cc], D50%[cGy], or CI between the clinically delivered plans without hippocampal dose objectives and the treatments replanned for meeting hippocampal dose constraints.

HD-MLC vs SD-MLC plans

HD-MLC vs SD-MLC Leaves on Hippocampal BED

Differentiation of the effects of SD-MLC leaves versus the Varian HD-MLC leaves is best done by comparing the two leaf sizes on plans without hippocampal optimization objectives and then separately comparing them on plans with hippocampal optimization objectives. Positive values of percent change signify an increase in a given metric.

**Figure 2:**
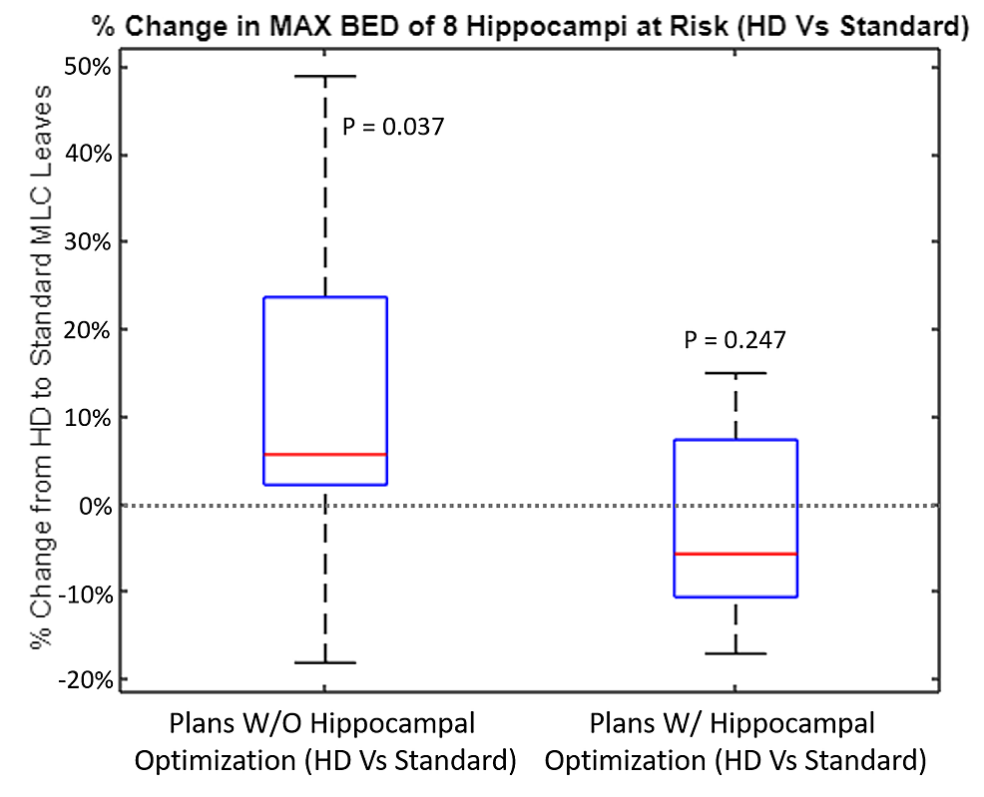
Percent Change in Max BED of Eight Hippocampi at Risk (HD vs Standard) This figure shows the average maximum BED values delivered across all eight hippocampi at risk across the seven patient plans being evaluated. The left side shows the effects of SD-MLCs on plans optimized without hippocampal objectives, whereas the right side shows the effect of SD-MLCs on plans optimized with hippocampal objectives. Only maximum BED is displayed because this was the only constraint that was exceeded in any of the initial 40 patient plans. BED, Biologically effective dose; SD, standard definition; HD, high definition; MLC, multileaf collimator

In the plans without hippocampal optimization objectives, the maximum BED exhibited an average increase of 11.65% ± 20.09% (p = 0.037) to the hippocampi at risk when SD-MLC leaves were employed versus HD-MLC leaves (Figure 2).

When plans were optimized for hippocampal avoidance, SD-MLC leaves had no statistically significant effect on the maximum BED to the hippocampus at risk (Figure 2). SD-MLC and HD-MLC leaves had the same success rate of meeting the hippocampal maximum BED constraint in the same five of eight hippocampi at risk. This is shown in Figure 1.

SD-MLC leaves resulted in increased BED across all 14 hippocampi in the seven patient plans being investigated, as stated in Tables 1, 2. The exception was in the plans that were optimized for hippocampal avoidance; the hippocampal maximum BED had no statistically significant change when using SD-MLC leaves (p = 0.75). This is likely due to the optimization objectives in these plans.

**Table 1:** Hippocampal BED: No Hippocampal Objectives This table displays the BED values of all 14 hippocampi across all seven plans being investigated. These values show the effects of SD leaves instead of HD leaves on plans without hippocampal optimization objectives. BED, Biologically effective dose; SD, standard definition; HD, high definition.

Hippocampal BED: No Hippocampal Objectives		HD (Gy)	SD (Gy)	% Change	p Value
						Mean BED	AVG	11.5	14.76	37.99%	0.01427
STD	9.08	10.65	37.53%								
Min BED	AVG	3.22	4.95	51.10%	0.0002
	STD	1.38	2.43	32.23%	
Max BED	AVG	50.65	56.95	33.76%	0.00457
	STD	67.82	71.12	58.38%	

**Table 2:** Hippocampal BED: With Hippocampal Objectives This table displays the BED values of all 14 hippocampi across all seven plans being investigated. These values show the effects of SD leaves instead of HD leaves in the plans that were optimized for hippocampal avoidance. BED, Biologically effective dose; SD, standard definition; HD, high definition.

Hippocampal BED: With Hippocampal Objectives		HD (Gy)	SD (Gy)	% Change	p Value
						Mean BED	AVG	8.89	10.5	27.88%	0.00004
STD	7.97	8.17	23.70%								
Min BED	AVG	2.96	3.86	36.91%	0.00401
	STD	1.25	1.28	34.61%	
Max BED	AVG	42.65	42.26	10.62%	0.74742
	STD	66.4	63.5	31.02%	

HD-MLC vs SD-MLC leaves on brain dose-volume data

**Figure 3:**
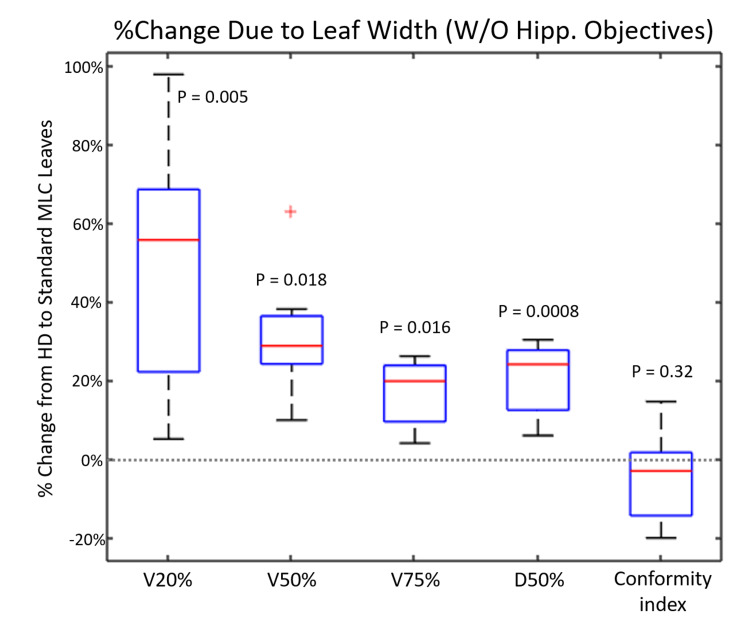
Percent Change Due to Leaf Width (Without Hippocampal Objectives) Change in dose-volume metrics when the SD-MLCs (5 mm) are used instead of HD-MLCs (2.5 mm) between plans without hippocampal objectives. SD, Standard definition; HD, high definition; MLC, multileaf collimator.

**Figure 4:**
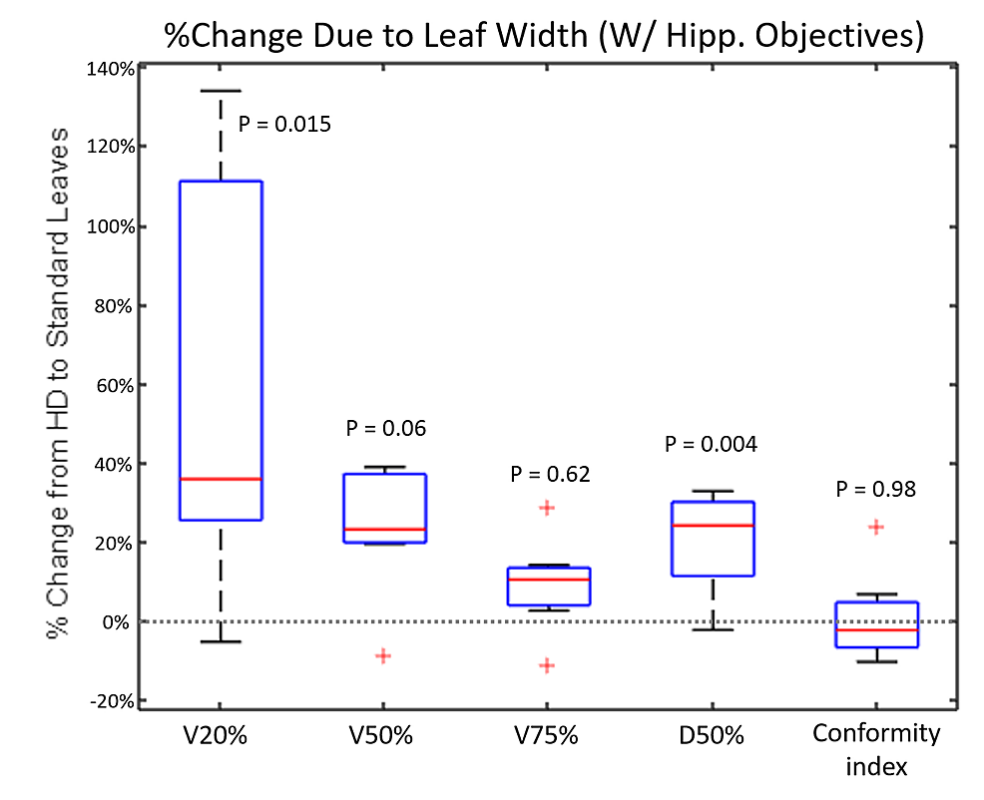
Percent Change Due to Leaf Width (With Hippocampal Objectives) Change in dose-volume metrics when the SD-MLCs (5 mm) are used instead of HD-MLCs (2.5 mm) between plans with hippocampal objectives. SD, Standard definition; HD, high definition; MLC, multileaf collimator.

In the plans without hippocampal optimization objectives, using SD-MLC leaves results in a statistically significant increase of the volume of healthy brain tissue receiving a given percentage of the prescribed dose (Figure 3). The most substantial change is in the V20%[CC] (volume receiving 20% of the prescribed dose) where the volume has an average percent increase of 48.22% ± 32.17% (p = 0.0056). As for the treatments replanned for reducing maximum hippocampal BED (Figure 4), the SD-MLC leaves also adversely impacted the volume of healthy brain tissue receiving a given dose, but this increase seemed to become less statistically significant for higher dose values (i.e., for V75%[cc] p = 0.642). For both the plans without hippocampal optimization objectives and the hippocampal avoidance plans, SD-MLC leaves had no statistically significant effect on the CI.

## Discussion

Out of the 40 patients considered in this study, seven had treatment plans that exceeded the RTOG 0933 hippocampal constraint for maximum BED, resulting in eight total hippocampi at risk. Five of the eight hippocampi at risk met BED constraints through small adjustments in the optimization process, whereas three did not. The ability to successfully meet dose constraints correlated with the distance between the nearest PTV and the hippocampus at risk. Successful hippocampal avoidance required at least 0.45 cm between the hippocampus and the nearest PTV, whereas the unsuccessfully avoided hippocampi either touched or overlapped with the nearest PTV (as shown in Figure 1). These findings were consistent with previous work evaluating the feasibility and risk factors in hippocampal avoidance for single-fraction SIMT treatments [17]. In this study, 10 out of 12 patient plans that risked exceeding hippocampal BED constraints had met these constraints through additional optimization [17]. This study also acknowledged the relationship between hippocampal BED and the distance between the hippocampus and the nearest target [17]. Neither study noticed significant tradeoffs between hippocampal optimization and changes in dose-volume measurements in healthy tissue [17].

Using the SD-MLC leaves for plans without hippocampal optimization objectives increased the volume of normal brain tissue receiving a given dose and increased the mean dose across all the hippocampi in all treatments. However, SD-MLC leaves did not result in an increase in the number of hippocampi that exceeded the maximum BED constraint.

The five of eight at-risk hippocampi that met BED constraints through optimization adjustments with HD-MLC leaves also met BED constraints with SD-MLC leaves post optimization. This reinforces that meeting hippocampal BED constraints is more dependent upon the distance between the hippocampus and the nearest target than it is on MLC leaf width. Additionally, SD-MLC leaves had no statistically significant change to the conformity of the treatments. This was true for both plans with and without hippocampal optimization objectives. However, in both cases there were increases in the volumes of healthy tissue receiving a given dose due to leaf width. Use of SD-MLC leaves resulted in a 24% ± 17% V50%[cc] increase in plans optimized for hippocampal constraints (p = 0.076) and 31% ± 16% in the plans without hippocampal constraints (p = 0.024). These findings are consistent with the findings from Abisheva et al. that also evaluated the effects of SD-MLC leaves on SIMT treatments, which found a 20.04% ± 0.13% increase in V50%[cc] [16]. Based on these results, it would be reasonable to speculate that there are a group of patients with brain metastases close to the hippocampi that could particularly benefit from the use of a system with the HD-MLCs. However, testing this hypothesis would require a far greater number of patients than were enrolled in this study.

## Conclusions

The work in this study shows that for patients receiving SIMT using VMAT with four to 10 metastases, the hippocampus may be at risk of dose exceeding the limits from RTOG 0933. For many of these patients, reducing hippocampal dose may be possible, granted sufficient distance between the hippocampus and the nearest PTV. Similar results in hippocampal avoidance can be obtained using SD-MLC leaves; however, there is a resulting increase in dose to healthy brain tissue elsewhere. Further research should include evaluation of the hippocampal dose across all 40 patients as a function of distance to the nearest PTV and considerations of the effects of varying PTV volumes. Additionally, a multi-institutional study should be conducted with a larger number of patients to determine if hippocampal avoidance significantly improves patient outcomes, not only in median survival but also in the preservation of neurocognition and QOL.
